# 560. Efficacy and Safety of Retavibart, a Long-acting Monoclonal Antibody, for the Prevention of Respiratory Syncytial Virus-Induced Lower Respiratory Tract Infection: A Phase 2b Trial in Infants Entering Their First Epidemic Season

**DOI:** 10.1093/ofid/ofaf695.169

**Published:** 2026-01-11

**Authors:** Yanyan Li, Daiyin Tian, Lingli Pan, Hanmin Liu, Enmei Liu, Shiru Zhao, Haiyan An, Xin Zhou, Jiyuan Ding, Jianli Chu, Meizhuo Zhang, Ying Wang, Xiaohu Kuang, Xia Zhou, Zhenxing Jia, Hao Zhao, Huaxin Liao, Wanmei wang

**Affiliations:** Zhuhai Trinomab Pharmaceutical Co., Ltd., Beijing, Beijing, China; Children's hospital of Chongqing Medical University, Chongqing, Chongqing, China; West China Second University Hospital, Chengdu, Sichuan, China; West China Second University Hospital, Chengdu, Sichuan, China; Children's Hospital of Chongqing Medical University, Chongqing, Chongqing, China; Zhuhai Trinomab Pharmaceutical Co., Ltd., Beijing, Beijing, China; Zhuhai Trinomab Pharmaceutical Co., Ltd., Beijing, Beijing, China; Zhuhai Trinomab Pharmaceutical Co., Ltd., Beijing, Beijing, China; Zhuhai Trinomab Pharmaceutical Co., Ltd., Beijing, Beijing, China; Zhuhai Trinomab Pharmaceutical Co., Ltd., Beijing, Beijing, China; Zhuhai Trinomab Pharmaceutical Co., Ltd., Beijing, Beijing, China; Zhuhai Trinomab Pharmaceutical Co., Ltd., Beijing, Beijing, China; Zhuhai Trinomab Pharmaceutical Co., Ltd., Beijing, Beijing, China; Zhuhai Trinomab Pharmaceutical Co., Ltd., Beijing, Beijing, China; Zhuhai Trinomab Pharmaceutical Co., Ltd., Beijing, Beijing, China; Zhuhai Trinomab Pharmaceutical Co., Ltd., Beijing, Beijing, China; Zhuhai Trinomab Pharmaceutical Co., Ltd., Beijing, Beijing, China; Zhuhai Trinomab Pharmaceutical Co., Ltd., Beijing, Beijing, China

## Abstract

**Background:**

Respiratory syncytial virus (RSV) is the most common cause of lower respiratory tract infection in young children. Retavibart (formerly known as TNM001) is a recombinant human monoclonal antibody (mAb) targeting the prefusion protein of RSV.

**Figure:**
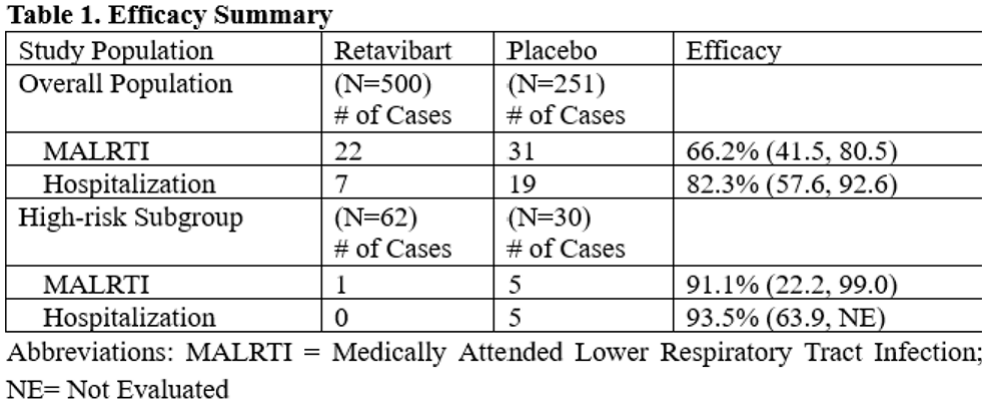

**Figure:**
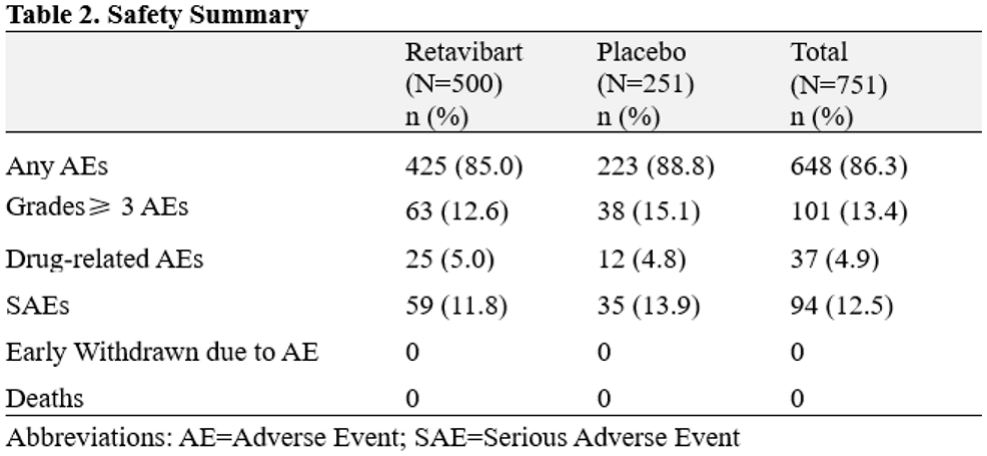

**Methods:**

This was a randomized, double-blind, placebo-controlled phase 2b trial. Eligible participants included term and preterm infants, with or without congenital heart disease (CHD) or chronic lung disease (CLD) of prematurity, who were entering their first RSV season. Participants were randomized in a 2:1 ratio to receive a single intramuscular injection of retavibart 120 mg or placebo. Efficacy endpoints included incidence of medically attended lower respiratory tract infection (MALRTI) and hospitalization due to RT-PCR confirmed RSV infection through Day 151. Safety assessments included the collection of adverse events (AEs) and serious adverse events (SAEs) and monitoring of vital signs within 240 days post-dose.

**Results:**

A total of 760 participants were randomized and 751 received treatment: 500 received retavibart and 251 received placebo. The median age of participants was 2.50 months (range: 0-8.1 months) with a slight male predominance. Ninety-two participants were classified as high-risk, including 65 healthy preterm infants (gestation age < 35 weeks) and 28 with CHD. No infants with CLD were enrolled.

Retavibart reduced the incidence of MALRTI by 66.2% (95% CI: 41.5, 80.5) and RSV-related hospitalization by 82.3% (95% CI: 57.6, 92.6) compared to placebo. Greater efficacy was observed in the high-risk population. In this subgroup, the reduction in RSV-related MALRTIs and hospitalization reached 91.1% and 93.5%, respectively.

The incidences of any AEs and SAEs were comparable between the two groups. No participants died or were early withdrawn from the trial due to AE.

**Conclusion:**

A single dose injection of retavibart 120 mg was well tolerated and had a safety profile comparable to that of placebo. Retavibart showed effective protection against RSV infection in infants during their first season. This was the first clinical trial that compared a long-acting anti-RSV mAb with placebo in the high-risk population. Results showed that retavibart had greater efficacy in this population.

**Disclosures:**

All Authors: No reported disclosures

